# Complete Response to Tislelizumab in an Elderly Patient With Metastatic Pulmonary Squamous Cell Carcinoma: Potential Synergy With Metformin and Multidisciplinary Management

**DOI:** 10.1002/ccr3.73481

**Published:** 2026-09-06

**Authors:** Lei Li

**Affiliations:** ^1^ Cancer Hospital of Shantou University Medical College Shantou Guangdong China

**Keywords:** case report, complete response, immunotherapy, metformin, multidisciplinary team (MDT), pulmonary squamous cell carcinoma, tislelizumab

## Abstract

Carefully selected elderly patients with metastatic PSCC may obtain a durable complete response via tislelizumab chemoimmunotherapy combined with metformin and standardized MDT management, providing practical clinical reference for high‐risk elderly lung cancer patients complicated with multiple comorbidities.

## 1 Introduction

Treating advanced pulmonary squamous cell carcinoma (PSCC) in elderly patients with comorbidities remains a significant clinical challenge worldwide [1]. Immunotherapy with anti‐PD‐1 antibodies such as tislelizumab combined with chemotherapy has become a standard first‐line strategy for advanced PSCC, substantially improving patient overall survival and progression‐free survival [2, 3]. However, complete response (CR) is rare in clinical practice, especially in elderly individuals with high tumor burden and multiple visceral metastases, and relevant clinical experience is limited.

This case has prominent clinical novelty and reference value. First, CR was achieved in a 72‐year‐old elderly patient with stage IVb PSCC complicated with multiple liver metastases, which is an uncommon clinical outcome. Second, the patient had long‐term Type 2 diabetes and continuously took metformin throughout anti‐tumor treatment; this case provides real‐world evidence for the potential synergistic effect of metformin and PD‐1 inhibitor [4]. Third, relying on standardized multidisciplinary team (MDT) management, we successfully maintained treatment tolerance in this elderly patient with hypertension and diabetes, offering a practical treatment model for elderly tumor patients with multiple comorbidities [5]. This case reports a sustained CR in an elderly male with high‐risk metastatic PSCC following chemoimmunotherapy, exploring the roles of effective treatment, multidisciplinary team (MDT) management, and potential adjunctive metformin.

Herein, we report this special case and analyze the treatment efficacy, safety, and relevant influencing factors in detail.

## 2 Case Presentation/Examination

A 72‐year‐old male with a 50 pack‐year smoking history presented in August 2024 with persistent cough and unintentional weight loss. His past medical history included chronic hypertension and Type 2 diabetes mellitus, which were stably managed with oral metformin (850 mg twice daily) and gliclazide for years. Baseline Eastern Cooperative Oncology Group (ECOG) performance status was 1.

**FIGURE 1:**
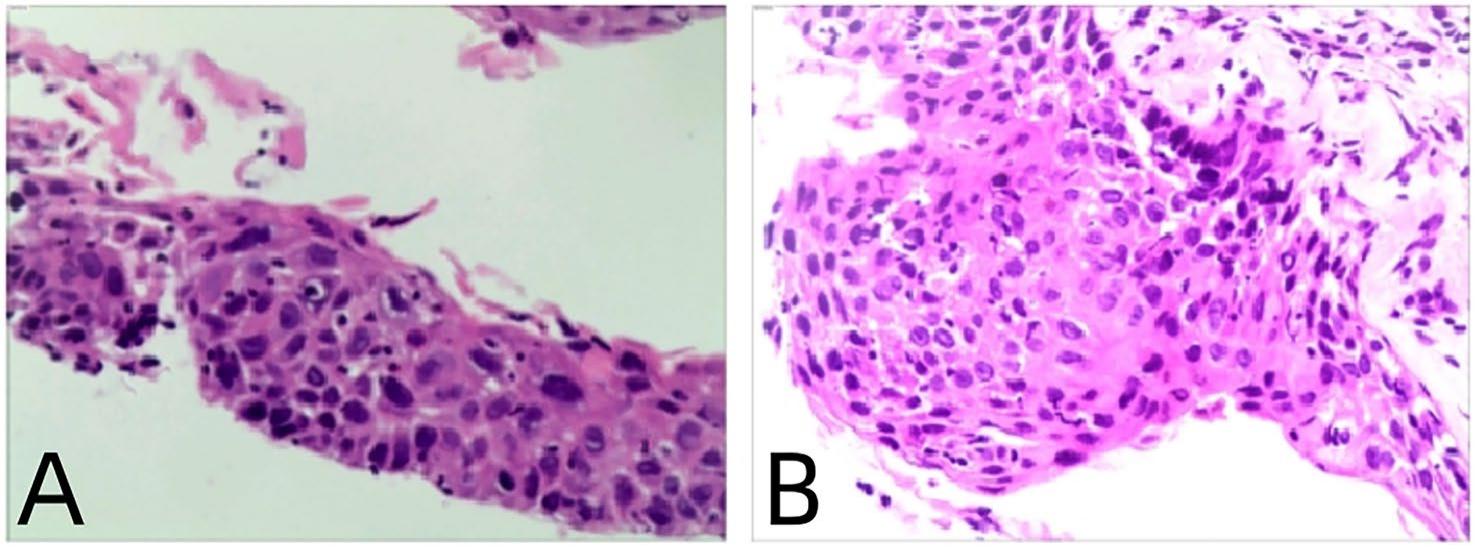
Histopathological micrograph of transthoracic lung biopsy specimen (lung parenchymal lesion tissue). Pathological diagnosis: Squamous cell carcinoma in situ; limited sampling cannot rule out invasive squamous cell carcinoma. (A) Low‐power magnification (×100) micrograph: Proliferative atypical squamous epithelial cells are strictly confined to the epithelial layer, with intact basement membrane and no obvious stromal invasion identified. (B) High‐power magnification (×400) micrograph: Severe nuclear atypia, irregular nuclear contours, elevated nuclear‐to‐cytoplasmic ratio, and complete loss of normal squamous epithelial stratification and polarity, morphologically consistent with squamous cell carcinoma in situ.

Imaging studies revealed a 4.8 cm space‐occupying lesion in the right lower lobe of the lung, combined with mediastinal lymphadenopathy and multiple space‐occupying lesions in the liver, which were diagnosed as stage IVb PSCC according to the 8th edition of AJCC lung cancer staging criteria [6]. Transthoracic needle biopsy confirmed moderately differentiated squamous cell carcinoma pathologically (Figure 1). Comprehensive molecular profiling via next‐generation sequencing was not performed due to extremely limited biopsy tissue. In addition, PSCC has inherently low prevalence of actionable driver mutations; meanwhile, the patient refused liquid biopsy for circulating tumor DNA detection, which further restricted molecular subtyping analysis. Serum squamous carcinoma biomarker Cyfra 21‐1 was obviously elevated at baseline.

## 3 Methods

All diagnostic, staging, and therapeutic procedures implemented for this patient are summarized within this unified Methods section without subheadings of differential diagnosis, investigations, and treatment, as requested by the reviewer.

Multiple layered examinations were conducted sequentially to complete differential diagnosis, definitive pathological typing, tumor staging, and baseline physical function evaluation for this elderly patient. Chest and abdominal contrast‐enhanced computed tomography (CT) was initially performed to characterize the primary lung lesion, mediastinal lymph node involvement, and hepatic metastatic lesions; these baseline CT images served as the reference standard for subsequent serial response assessments per RECIST v1.1 criteria. Transthoracic percutaneous lung biopsy was arranged under CT guidance to acquire pathological tissue specimens for histological identification, which excluded alternative malignant diagnoses including primary lung adenocarcinoma and small cell lung cancer based on characteristic squamous epithelial morphology, absent glandular differentiation, and lack of primitive small undifferentiated tumor cells with brisk mitoses. Systemic serum biomarker testing for Cyfra 21‐1, a specific serological marker for squamous cell carcinoma, was scheduled at baseline and repeated throughout all treatment cycles to dynamically track tumor burden fluctuations. Comprehensive geriatric assessment was also performed, including ECOG performance status scoring and structured evaluation of chronic hypertension and Type 2 diabetes, to quantify the patient's physical reserve and predict tolerance to combined chemoimmunotherapy.

After unified MDT consensus discussion integrating medical oncology, radiology, pathology, geriatric internal medicine, and endocrine specialists, the first‐line induction regimen was determined as tislelizumab 200 mg intravenous infusion every 3 weeks combined with nab‐paclitaxel 260 mg/m^2^ and carboplatin AUC 5 administered on day 1 of each 21‐day cycle for a planned total of four induction cycles. The patient's home anti‐diabetic regimen of twice‐daily metformin 850 mg was maintained without interruption during all anti‐tumor treatment periods to sustain stable glycemic control and explore potential immunostimulatory synergy with PD‐1 blockade. Following completion of four cycles of combination induction therapy, single‐agent tislelizumab maintenance immunotherapy at the identical 200 mg every 3‐week dosage was continued long‐term until confirmed complete response.

## 4 Results and Conclusion

This section integrates all treatment response, safety, and long‐term follow‐up outcomes without separate subsections for Results and Follow‐up. Multiple descriptive paragraphs are included as required.

After completion of four cycles of induction chemoimmunotherapy, restaging contrast‐enhanced chest and abdominal CT demonstrated a partial treatment response: marked volumetric shrinkage of the primary right lower lobe pulmonary lesion and near complete resolution of all measurable liver metastatic lesions. Concurrent serum Cyfra 21‐1 concentrations exhibited an obvious downward trend, consistent with radiological tumor regression. No dose delays, dose reductions, or permanent treatment discontinuations were required during induction therapy, and no grade ≥ 3 immune‐related adverse events or chemotherapy‐associated toxicities were observed; blood pressure and blood glucose levels remained controllable under continuous combined internal medicine and endocrine monitoring throughout this treatment phase.

**FIGURE 2:**
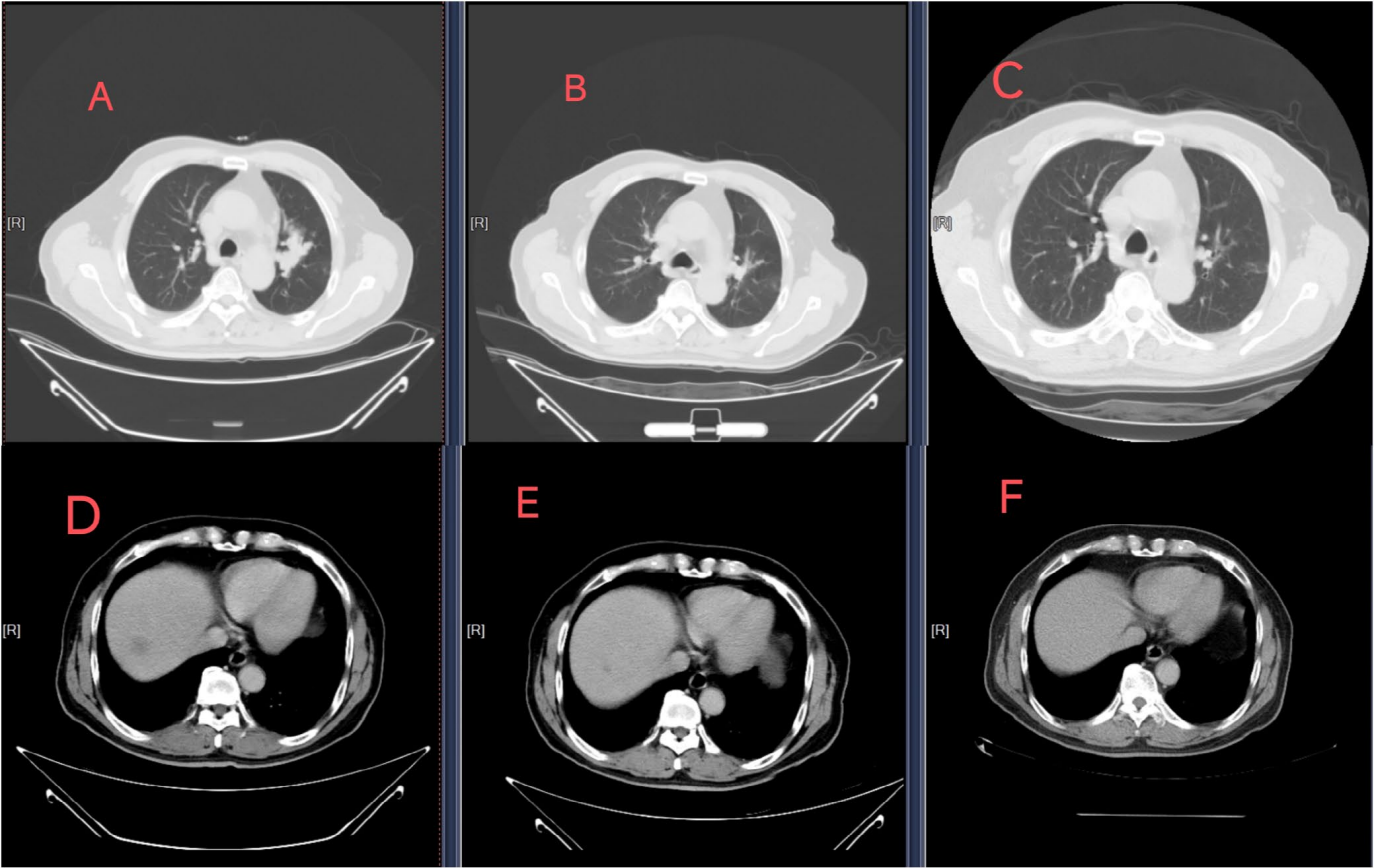
Imaging comparison of target lesions before and after treatment. (A) Baseline CT image of primary lung lesion (August 2024); (B) Lung lesion after 2 cycles of combined treatment; (C) Lung lesion after 11 cycles of tislelizumab‐based treatment; (D) Baseline CT image of liver metastatic lesions; (E) Liver metastatic lesions after 2 cycles of combined treatment; (F) Liver metastatic lesions after 11 cycles of tislelizumab‐based treatment. All lesions achieved complete regression at the final follow‐up.

**FIGURE 3:**
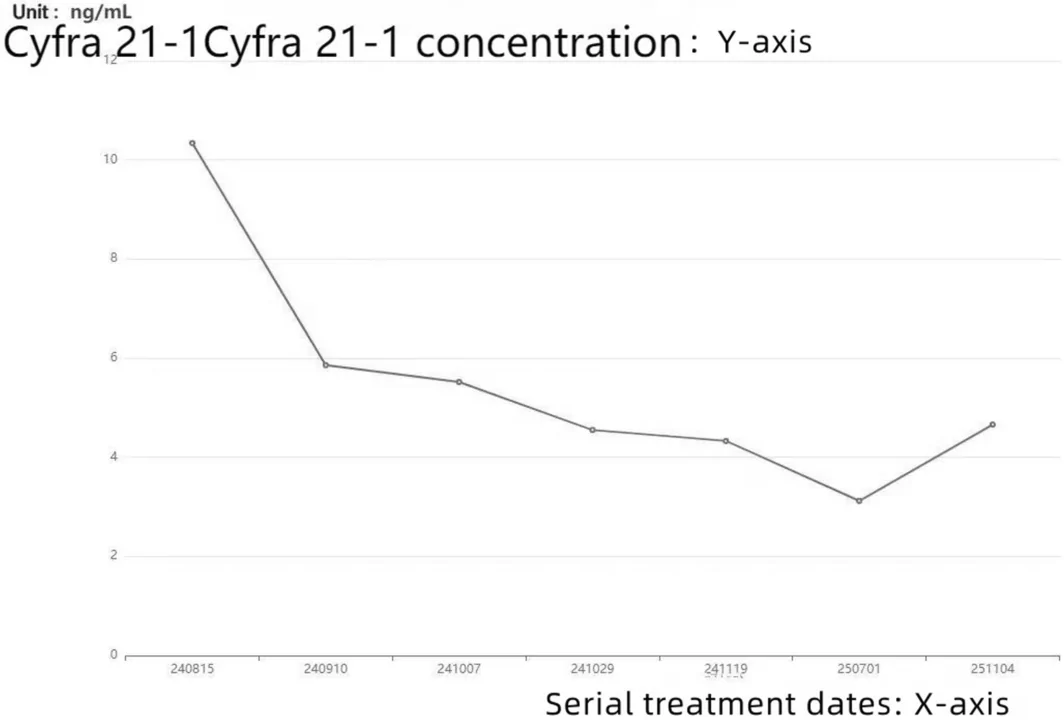
Dynamic trend of serum Cyfra 21‐1 levels during the whole treatment period. Vertical axis (*Y*‐axis): Serum Cyfra 21‐1 concentration, unit: ng/mL; Horizontal axis (*X*‐axis): Serial treatment dates formatted as YYYYMMDD. The biomarker gradually declined and returned to the normal range as treatment proceeded.

Following seven additional cycles of tislelizumab single‐agent maintenance therapy (11 total cycles of PD‐1 inhibitor treatment), repeat cross‐sectional imaging and serological testing fulfilled all RECIST v1.1 criteria for confirmed complete response (CR), with full disappearance of all target pulmonary and hepatic lesions documented on CT scans (Figure 2). Serum Cyfra 21–1 levels normalized completely and remained persistently within the reference normal range across all subsequent follow‐up visits (Figure 3). This sustained complete antitumor response has been maintained for more than eight consecutive months at the time of manuscript submission. The patient preserved stable ECOG performance status 1 throughout the entire treatment and surveillance period, retaining independent daily living ability without symptomatic deterioration related to underlying malignancy or chronic comorbidities.

This exploratory single‐case observation indicates that carefully selected elderly metastatic PSCC patients with well‐controlled chronic comorbidities and preserved physical function can achieve durable complete tumor regression with tislelizumab‐based first‐line chemoimmunotherapy. Concurrent maintenance of metformin may provide auxiliary immunomodulatory synergistic effects to strengthen PD‐1 inhibitor antitumor activity, while standardized interdisciplinary MDT care is indispensable to sustain treatment tolerability and maximize long‐term therapeutic benefit for this vulnerable geriatric patient subgroup. Importantly, this combined therapeutic strategy cannot be universally extrapolated to all elderly PSCC patients with visceral metastasis, and large‐scale prospective clinical trials are necessary to validate the reproducibility of these favorable clinical outcomes.

## 5 Discussion

This case highlights a durable CR in an elderly high‐risk PSCC patient, with several key contributing factors. Tislelizumab, engineered to minimize FcγR binding, has established reliable efficacy in PSCC according to the RATIONALE‐307 trial, and the patient's heavy smoking history likely increased tumor mutational burden, enhancing immunotherapy responsiveness [7]. Concomitant metformin may have synergized via immunomodulatory mechanisms (e.g., PD‐L1 degradation, tumor microenvironment modulation) supported by multiple preclinical and clinical evidence in non‐small cell lung cancer [8, 9].

Published data from Phase III clinical trials showed that tislelizumab plus chemotherapy significantly improved objective response rate in advanced PSCC patients, but CR rate was still low, especially in elderly patients with visceral metastasis. Most existing case reports about metformin combined with immune checkpoint inhibitors focused on efficacy improvement and drug resistance reversal in lung cancer, while reports on sustained CR in elderly metastatic PSCC are scarce [10, 11]. Compared with previous studies, our case further supplements the real‐world efficacy of this combined regimen in elderly patients with multiple comorbidities.

The MDT's role was pivotal: formulating age‐appropriate, evidence‐based therapy, managing comorbidities (hypertension, diabetes) to maintain treatment tolerance, and proactively monitoring for immune‐related adverse events [12]. This holistic approach ensured treatment continuity, a key determinant of immunotherapy success.

It should be noted that the current follow‐up duration of this case is only more than 8 months, which is relatively short. Long‐term durability of CR and long‐term safety of continuous combined medication cannot be fully evaluated. We will continue long‐term follow‐up for this patient to observe long‐term prognosis. Notably, this case supports immunotherapy use in carefully assessed elderly patients, emphasizing fitness and comorbidity control over chronological age [13, 14]. The potential synergy between metformin and immune checkpoint inhibitors warrants further large‐sample prospective clinical investigation in PSCC.

## Author Contributions

**Lei Li:** conceptualization, data curation, writing – original draft.

## Funding

The author has nothing to report.

## Consent

Written informed consent was obtained from the patient for publication of this case report and any accompanying images. A copy of the written consent is available for review by the Editor‐in‐Chief of this journal.

## Conflicts of Interest

The author declares that there are no conflicts of interest related to this case report. No financial support, consultancy fees, stock ownership, or institutional research grants were received from pharmaceutical manufacturers, biotech companies, or other commercial entities involved in the production or marketing of tislelizumab, nab‐paclitaxel, carboplatin, or metformin. All clinical observations, data collection, and manuscript writing were completed independently without external commercial influence.

## Data Availability

All de‐identified clinical datasets supporting the findings of this case report, including serial CT imaging measurements, longitudinal Cyfra 21‐1 laboratory values, treatment cycle records, pathological biopsy reports, and safety monitoring logs, are fully contained within the manuscript text, figures, and figure legends. Additional raw de‐identified patient source data (high‐resolution pathological slides, full uncropped CT scan images, complete lab test reports) are stored securely on the institutional encrypted hospital server. Qualified editors and peer reviewers may request access to these raw datasets by contacting the corresponding author via institutional email stuzlyyrsk@163.com; data access will be granted upon confirmation of formal peer review identity, subject to institutional patient privacy protection regulations.
